# *Sall1* in renal stromal progenitors non-cell autonomously restricts the excessive expansion of nephron progenitors

**DOI:** 10.1038/srep15676

**Published:** 2015-10-29

**Authors:** Tomoko Ohmori, Shunsuke Tanigawa, Yusuke Kaku, Sayoko Fujimura, Ryuichi Nishinakamura

**Affiliations:** 1 Department of Kidney Development, Institute of Molecular Embryology and Genetics, Kumamoto University, 2-2-1 Honjo, Kumamoto 860-0811, Japan; 2 Program for leading graduate schools, HIGO program, Kumamoto University, 2-2-1 Honjo, Kumamoto 860-0811, Japan; 3 Liaison Laboratory Promotion Facility, Institute of Molecular Embryology and Genetics, Kumamoto University, 2-2-1 Honjo, Kumamoto 860-0811, Japan

## Abstract

The mammalian kidney develops from reciprocal interactions between the metanephric mesenchyme and ureteric bud, the former of which contains nephron progenitors. The third lineage, the stroma, fills up the interstitial space and is derived from distinct progenitors that express the transcription factor Foxd1. We showed previously that deletion of the nuclear factor *Sall1* in nephron progenitors leads to their depletion in mice. However, *Sall1* is expressed not only in nephron progenitors but also in stromal progenitors. Here we report that specific *Sall1* deletion in stromal progenitors leads to aberrant expansion of nephron progenitors, which is in sharp contrast with a nephron progenitor-specific deletion. The mutant mice also exhibited cystic kidneys after birth and died before adulthood. We found that *Decorin*, which inhibits Bmp-mediated nephron differentiation, was upregulated in the mutant stroma. In contrast, the expression of *Fat4*, which restricts nephron progenitor expansion, was reduced mildly. Furthermore, the Sall1 protein binds to many stroma-related gene loci, including *Decorin* and *Fat4*. Thus, the expression of *Sall1* in stromal progenitors restricts the excessive expansion of nephron progenitors in a non-cell autonomous manner, and *Sall1*-mediated regulation of *Decorin* and *Fat4* might at least partially underlie the pathogenesis.

A typical mammalian kidney contains approximately one million nephrons, which are functional units consisting of glomeruli, proximal and distal renal tubules, and collecting ducts. During development, the nephron is formed by reciprocally inductive interactions between two precursor tissues: the metanephric mesenchyme and the ureteric bud. The former contains nephron progenitors that express the transcription factor Six2, and give rise to most components of the nephron epithelia, including those in glomeruli (podocytes and parietal cells) and renal tubules^1^. In contrast, the collecting duct epithelium is derived from the ureteric bud. However, these two lineages are not sufficient to generate the complete kidney structure. A third lineage—the stroma—is required, which is derived from a distinct progenitor population that expresses the transcription factor Foxd1^2^,^3^. The stromal progenitors surround the Six2-positive nephron progenitors, and are maintained in the outermost cortical region of the kidney during development. The interstitial tissue between the nephron’s epithelia is filled with differentiated stromal cells, such as fibroblasts, pericytes, and glomerular mesangial cells, the latter two being closely associated with vasculature.

The stromal cells not only fill up the interstitial space but also regulate the development of the nephron. Several genes for transcription factors are expressed in the cortical stroma, including *Foxd1, Pbx1*, and *Tcf21* (also known as *Pod1*). Deletion of these genes in mice results in nephron progenitor expansion^4^,^5^,^6^,^7^,^8^, suggesting an interaction between the stroma and the nephron. It has been reported that *Foxd1* deletion leads to *Decorin* up-regulation^9^. *Decorin* encodes a proteoglycan that functions as a sequestering receptor for the Tgf-β family of ligands, thus the increase in *Decorin* expression inhibits Bmp-mediated nephron differentiation resulting in progenitor expansion. *Foxd1Cre*-mediated cell ablation also leads to nephron progenitor expansion^10^,^11^, and this phenotype is explained by the loss of *Fat4* in the stroma^10^. Fat4 belongs to an atypical cadherin family conserved between *Drosophila* and mammals, and stroma-specific *Fat4* deletion leads to nephron progenitor expansion^12^. Whereas the Fat proteins in *Drosophila* activate the Hippo pathway, it remains controversial whether *Fat4* deletion in the mammalian kidney stroma leads to a reduction of the Hippo pathway and subsequent nuclear localisation of Yap/Taz in nephron progenitors^10^,^12^,^13^. *Fat4* is also involved in planar cell polarity and ubiquitous *Fat4* deletion disrupts oriented cell division, leading to dilatation and impaired elongation of nephrons^14^. However, it remains unclear in which lineage a *Fat4* deletion is responsible for the phenotype.

The region-specific homeotic gene *spalt* (*sal*) was first isolated from *Drosophila* and encodes a nuclear protein characterised by multiple double zinc finger motifs^15^. Humans and mice each have four known *sal*-*like* genes. Mutations in human *SALL1* and *SALL4* have been associated with Townes–Brocks and Okihiro syndromes, respectively, both of which are autosomal dominant diseases that involve abnormalities in various organs including ears, limbs, heart, and kidneys^16^,^17^. We have shown that conventional *Sall1* knockout mice exhibit kidney agenesis resulting from failure of ureteric bud attraction toward the mesenchyme^18^. *Sall1*-expressing cells in the metanephric mesenchyme represent multipotent nephron progenitors^19^, and *Sall1* deletion in *Six2*-positive nephron progenitors results in kidney hypoplasia and severe progenitor depletion^20^. Thus, *Sall1* is essential for the maintenance of nephron progenitors. However, *Sall1* is not only expressed in nephron progenitors but also in stromal progenitors^20^. To show the role of *Sall1* in the latter cell population, we deleted *Sall1* in stromal progenitors by utilizing the *Foxd1Cre* strain^2^,^3^. Although *Foxd1* is also expressed in developing podocytes, *Foxd1Cre* is detected only in a subset of maturing-stage podocytes^2^,^21^. In addition, *Sall1* expression in podocytes is minimal. Therefore, we reasoned that we could address the role of *Sall1* in stromal progenitors through the analysis of *Foxd1Cre:Sall1*^*flox/flox*^ mice.

## Results

### Mice lacking *Sall1* in the stromal progenitors exhibit dilatation of all nephron components and die before adulthood

**Figure 1:**
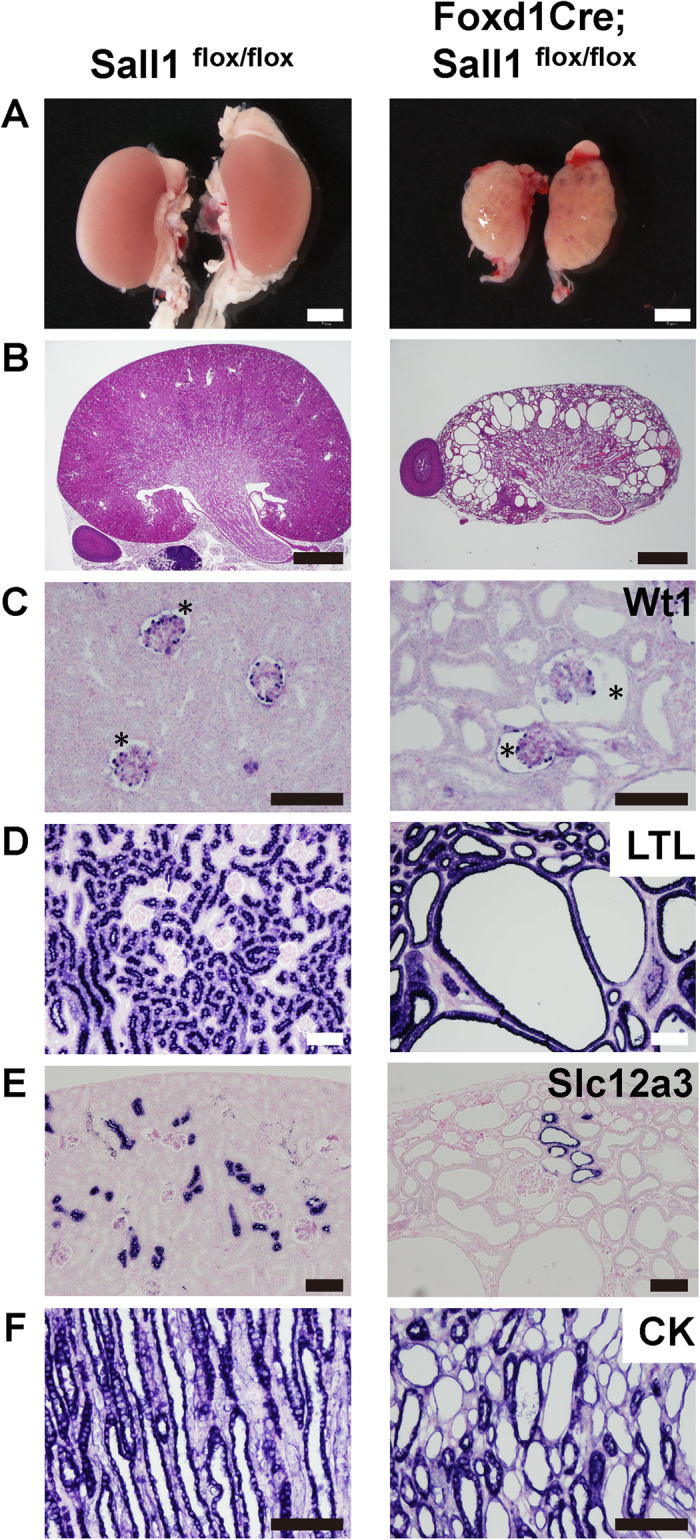
Mice lacking *Sall1* in the stromal progenitors exhibit dilatation of all nephron components. (**A**) Macroscopic views of *Sall1*^*flox/flox*^ and *Foxd1*Cre; *Sall1*^*flox/flox*^ kidneys at 8 weeks of age, showing a significant reduction in size of the mutant kidney. (**B**) Haematoxylin and eosin (HE) staining of control and mutant kidneys at 8 weeks of age. (**C**) Expansion of Bowman’s space (asterisks). Glomerular podocytes have been stained with an anti-Wt1 antibody. (**D–F**) Dilatation of proximal tubules (LTL immunostaining), distal tubules (Slc12a3), and collecting ducts (cytokeratin, CK). Scale bars: (A) = 2 mm; (B) = 1 mm; (C–F) = 100 μm.

To examine the roles of *Sall1* in the stroma, we crossed the floxed allele of *Sall1* with *Foxd1Cre* mice expressing Cre recombinase in their stromal progenitors^3^,^20^. The *Foxd1Cre; Sall1*^*flox/flox*^ mice were born alive but most of them had died before 5 weeks of age (n = 8 out of 10). The mutant kidney was reduced in size, and contained multiple cysts (Fig. 1A,B). Staining of the markers for various nephron lineages showed that all the nephron components were dilated, including Bowman’s space of the glomeruli, *Lotus tetragonolobus* lectin (LTL)-positive proximal and Slc12a3-positive distal tubules, and cytokeratin-positive collecting ducts (Fig. 1C–F). The surviving mutants also appeared sick and showed similar kidney phenotypes, when euthanized at 8 weeks after birth (n = 2). Consistent with the histology, the renal functions of these two mice were worse than their littermates, as shown by the elevated levels of blood urea nitrogen (290.0 and 43.8 mg/dl in the mutants vs. 30.7 and 26.2 mg/dl in the controls), and of serum creatinine (1.00 and 0.18 mg/dl in the mutants vs. 0.11 and 0.12 mg/dl in the controls). Because we never observed ureteric dilatations, these phenotypes were unlikely to be caused by obstruction of the lower urinary tract.

### Nephron progenitors are excessively expanded in the *Sall1*-deficient kidney

**Figure 2:**
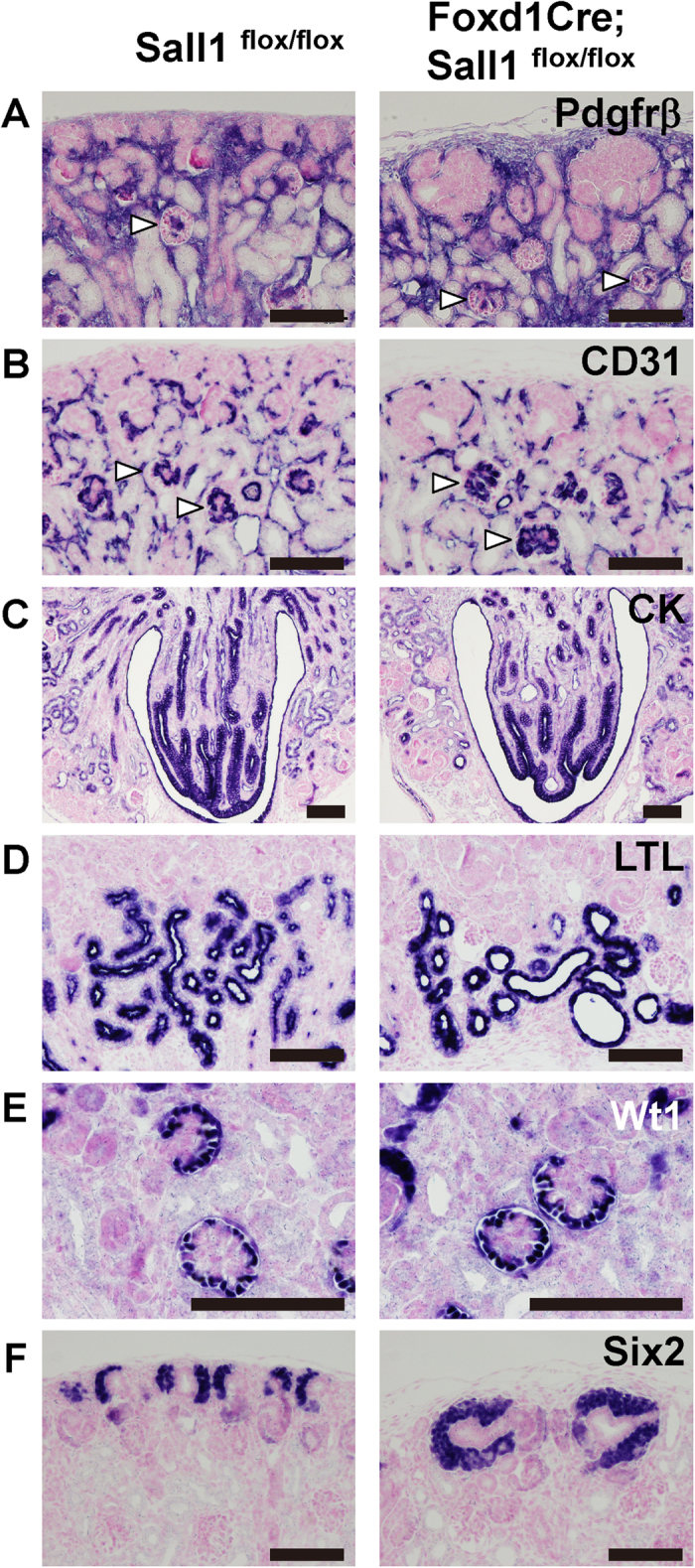
Nephrons start to dilate in *Sall1*-deficient neonatal mice. (**A,B**) Formation of stromal cells and vasculature is unaffected at birth. Immunostaining of Pdgfrβ (**A**) and CD31 (**B**), respectively. Arrowheads: glomeruli. (**C**) Collecting ducts in the medulla shown by immunostaining for cytokeratin. (**D**) Some proximal tubules are dilated, as shown by LTL immunostaining. (**E**) Bowman’s space of the glomeruli is not dilated yet. Glomerular podocytes are immunostained with an anti-Wt1 antibody. (**F**) Immunostaining for Six2 shows expansion of nephron progenitors at birth. Scale bars = 100 μm.

When analysed at birth, stromal cells, which are positive for platelet-derived growth factor receptor β (Pdgfrβ)^2^, were detected throughout the interstitial space in the mutants, as in the controls. Pdgfrβ-positive cells in the glomeruli—corresponding to mesangial cells—were also detectable in the mutants (Fig. 2A). Vascular endothelial cells were distributed in the interstitial space and in glomeruli (Fig. 2B). Thus, the development of stromal cells and vascular tissues were apparently unaffected in the *Sall1* mutant neonatal mice. The dilatations of the nephron were not yet prominent: the proximal tubules were mildly dilated, while no dilatations were observed in the other parts of the nephron, including Bowman’s space of the glomeruli (Fig. 2C–E). More remarkably, areas of Six2-positive nephron progenitors at birth were expanded and thickened around the ureteric bud tips (Fig. 2F). Therefore, the major abnormalities in the stroma-specific *Sall1* deletion were non-cell autonomously observed in the nephrons: namely nephron dilatation and progenitor expansion.

**Figure 3:**
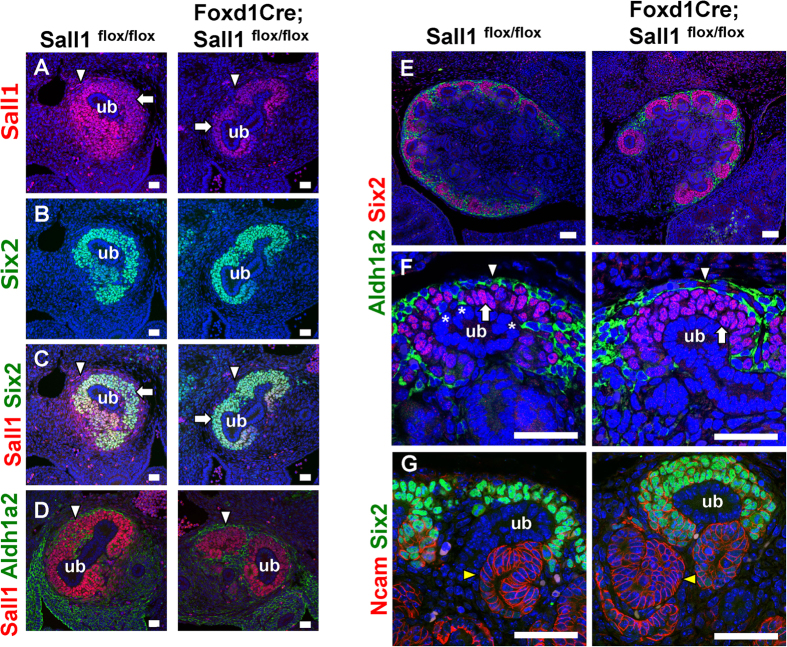
Nephron progenitors are expanded in the *Sall1* mutant kidney. (**A–C**) Dual immunostaining for *Sall1* (red) and Six2 (green) in *Sall1*^*flox/flox*^ and *Foxd1Cre; Sall1*^*flox/flox*^ kidneys at E12.5. *Sall1* expression in the stroma (arrowheads) is absent but is retained in the nephron progenitors (arrows) that are positive for Six2. ub: ureteric bud. (**D**) Immunostaining for *Sall1* (red) and Aldh1a2 (green) of *Sall1*^*flox/flox*^ and *Foxd1GFPCre; Sall1*^*flox/flox*^ kidneys at E12.5. Note the absence of *Sall1* in the Aldh1a2-positive stromal cells (arrows). (**E**,**F**) Dual immunostaining for Six2 (red) and Aldh1a2 (green) at E14.5. Six2-positive nephron progenitors (arrows) are expanded, while Aldh1a2-positive stromal cells (arrowheads) are retained in the mutant kidney. Some stromal cells (asterisks) reach the ureteric bud (ub) tip epithelium in the control. (**G**) Dual immunostaining for Six2 (green) and Ncam (red) at E14.5. Nephron progenitors are expanded, but the Ncam-positive nascent nephrons (yellow arrowheads) are formed. Scale bars = 20 μm.

Next, we analysed the mutant mice during gestation. At embryonic day (E) 12.5, *Sall1* was expressed not only in Six2-positive nephron progenitors but also in the surrounding stroma in the controls (Fig. 3A–C, Supplementary Fig. S1), as we reported previously^20^. *Sall1* expression in the stroma disappeared in the *Foxd1Cre; Sall1*^*flox/flox*^ kidney, and was restricted to the Six2-positive domain (Fig. 3A–C). Co-immunostaining for the stromal marker Aldh1a2 (also known as retinoic acid dehydrogenase 2: Raldh2)^22^ also showed the absence of *Sall1* in the stromal cells (Fig. 3D). Because *Foxd1Cre* mice express a fusion protein of Green Fluorescent Protein (GFP) and Cre recombinase, GFP was detected in the stromal progenitors surrounding the nephron progenitors, and *Sall1* was absent in the former population (Supplementary Fig. S1).

Thus, *Sall1* was deleted specifically in the stroma by E12.5. At this stage, nephron progenitor expansion was not apparent (Fig. 3B). The progenitor numbers per ureteric bud (the mean ± standard deviation) were 138.7 ± 25.8 in the mutants, while they were 124.4 ± 25.3 in the controls (p = 0.24). However, at E14.5, expansion of nephron progenitors was evident (Fig. 3E–G, Supplementary Fig. S1). The progenitor numbers per ureteric bud were 63.7 ± 18.5 in the mutants, while they were 41.7 ± 12.2 in the controls (p < 0.01). In the controls, Six2-positive nephron progenitors had accumulated around the ureteric bud tips, and Aldh1a2-positive stromal cells surrounded the nephron progenitors (Fig. 3E). Notably, some stromal cells penetrated the Six2-positive areas and directly attached to the ureteric bud epithelia (Fig. 3F), which might explain Ret regulation in the ureteric bud by retinoic acid supplied from the stroma^22^. In the *Sall1* mutants, the layers of Six2-positive cells were thickened, and the stromal cells rarely reached the ureteric buds. Differentiation of the nephrons was not affected significantly, because the morphology of Ncam-positive nascent nephrons was similar to that in the controls (Fig. 3G). Thus, nephron progenitor expansion started at mid-gestation upon stroma-specific *Sall1* deletion, which suggests that the non-cell autonomous restriction of nephron progenitors by the stroma is affected in the absence of *Sall1.*

In contrast, we did not detect any significant differences in cell deaths between the control and mutant stromal progenitors at E13.5 or E14.5 (Supplementary Fig. S2), although the mutant kidneys showed mild increases. Cell proliferation, as shown by staining for phosphorylated histone H3 (PHH3), also exhibited no differences (Supplementary Fig. S2). We then introduced a tdTomato lineage reporter into the *Sall1* mutant mice. In the controls, all the stromal cells in the interstitial space, as well as glomerular mesangial cells, were labelled with tdTomato (Supplementary Fig. S2), which is consistent with reports that these cells are derived from *Foxd1*-positive stromal progenitors^2^,^3^. In the *Sall1* mutants, the same population was labelled, and the signals were absent from nephron progenitors and the ureteric buds, as in the controls, suggesting that aberrant differentiation from the stroma toward the other lineages did not occur in the absence of *Sall1*.

### Fat4 is reduced and Decorin is increased in *Sall1* mutant kidneys

**Figure 4:**
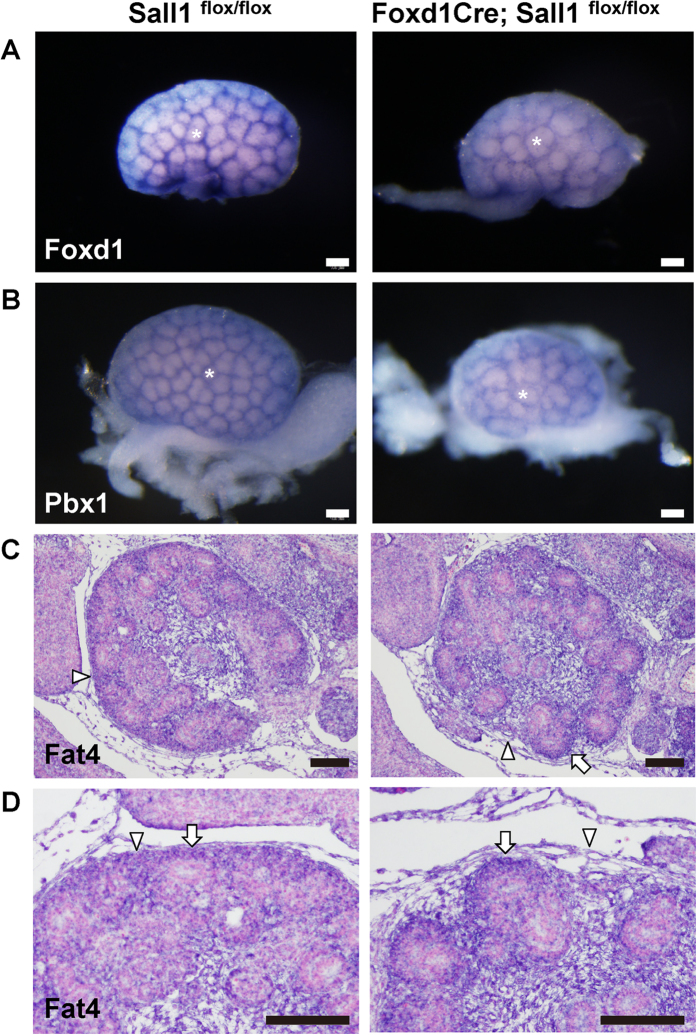
*Fat4* expression is reduced mildly in the *Sall1* mutant kidney. (**A,B**) Whole-mount *in situ* hybridisation of the control and *Sall1* mutant kidneys at E14.5. The expression levels of *Foxd1* (**A**) and *Pbx1* (**B**) are unaffected. The size of each nephron progenitor cluster (white asterisks) is increased in the mutant kidneys. (**C**,**D**) Section *in situ* hybridisation of *Fat4* at E14.5. Expression in the cortical stroma (arrowheads) is slightly reduced, but that in the nephron progenitors (arrows) is retained. (**D**) magnified images of panel (**C**). Scale bars = 100 μm.

Nephron progenitor expansion has been reported in several mutant mice lacking *Foxd1* or *Pbx1*^4^,^5^,^6^. These genes, as well as *Meis1*, produced mesh-like patterns in control embryonic kidneys as shown by whole mount *in situ* hybridisation (Fig. 4A,B; Supplementary Fig. S3). This was because they were more abundantly expressed in the cortical stroma filling the gaps between the clusters of nephron progenitors. None of these gene expression levels was decreased in the *Sall1* mutants. However, the meshes were larger than in the controls, which is consistent with the expansion of nephron progenitor clusters, as shown in Fig. 3. *Hoxd10*, expressed both in nephron progenitors and cortical stroma^23^, and *Tcf21*, expressed more abundantly in interstitial stroma^8^, were also unaffected in the *Sall1* mutants (Supplementary Fig. S3).

**Figure 5:**
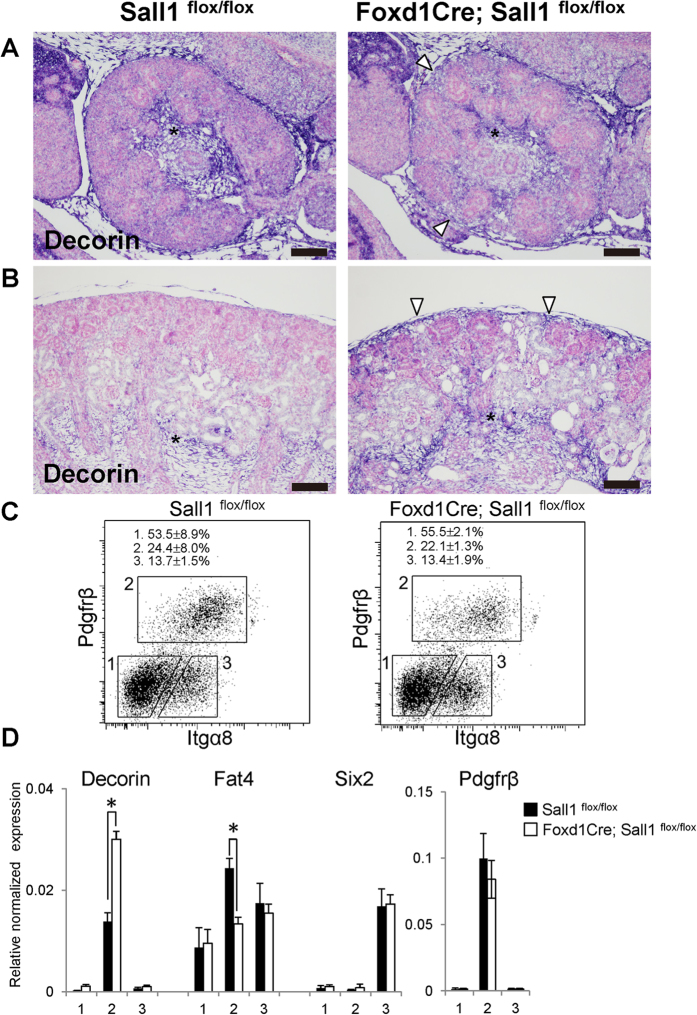
*Decorin* is increased in the *Sall1* mutant stromal cells. (**A**,**B**) Section *in situ* hybridisation of *Decorin* at E14.5 (**A**) and P0 (**B**). Expression domain is expanded from the medulla (asterisks) to the cortical region (arrowheads) in the mutant kidneys. (**C**) FACS analysis of control and mutant new-born kidneys. Fraction 1: Pdgfrβ−/integrin α8− cells, Fraction 2: Pdgfrβ+/integrin α8− stromal cells, Fraction 3: Pdgfrβ−/integrin α8+ nephron progenitors. The percentages of each fraction represent the average and standard deviation of *Sall1*^*flox/flox*^ controls and *Sall1*-deficient (*Foxd1GFPCre; Sall1*^*flox/flox*^) mutants (3 embryos each). (**D**) Quantitative RT-PCR analysis of the three fractions. The means and standard deviations of three controls and three mutants are shown. *p < 0.01 by Student’s *t* test. Scale bars = 100 μm.

Conventional and cortical stroma-specific deletion of *Fat4* in mice leads to nephron progenitor expansion^10^,^11^,^12^,^13^. It has also been reported that *Foxd1* deletion leads to *Decorin* up-regulation, which inhibits Bmp-mediated differentiation resulting in nephron progenitor expansion^9^. In the *Sall1* mutants, *in situ* hybridisation on sections showed that *Fat4* was slightly reduced in the cortical stroma but was retained in the nephron progenitors (Fig. 4C,D). Whereas *Decorin* was mainly expressed in the medullary stroma in the controls, its expression domain began to expand to the cortical region in the mutants at E14.5 (Fig. 5A), and became apparent at P0 (Fig. 5B).

To quantify the expression levels of *Decorin* and *Fat4*, we further sorted the *Pdgfrβ*-positive stromal cells from newborn kidneys (Fig. 5C). Whereas the percentages of stromal cells in the kidneys were similar between the controls and the *Sall1* mutants, our data showed that *Decorin* was upregulated in the mutant stroma, while *Fat4* expression was reduced mildly (Fig. 5D). Thus, misregulation of *Decorin* and *Fat4* might be responsible—at least partially—for nephron progenitor expansion in the *Sall1* mutants. Six2 expression in the nephron progenitor fraction, which was sorted based on integrin α8 expression^24^, was unaltered in the mutants, which is consistent with the immunostaining data in Fig. 3.

### *Sall1* binds directly to many stroma-related gene loci, including Decorin and Fat4

**Figure 6:**
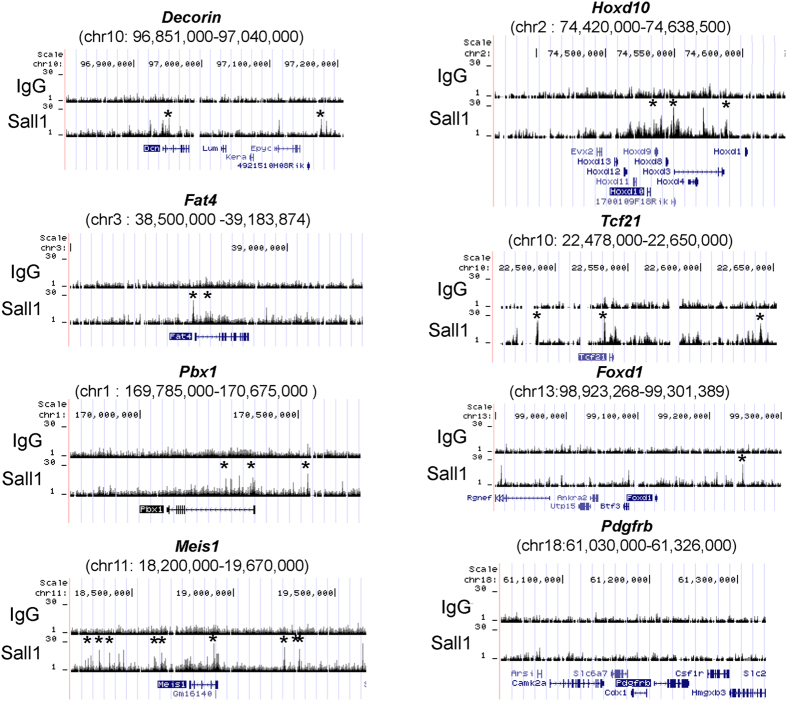
*Sall1* binds directly to stroma-related gene loci, including *Decorin* and *Fat4*. ChIP-seq analysis of *Sall1* within the stromal progenitor-related loci (mm9 coordinates). *Sall1* binds to *Decorin, Fat4*, *Pbx1*, *Meis1*, *Hoxd10*, and *Tcf21* loci, but not to *Foxd1* or *Pdgfr*β loci. Asterisks show peaks occupied by *Sall1*. Peak calling was carried out as described^20^.

We previously performed chromatin immunoprecipitation-sequencing (ChIP-seq) analysis by using the whole embryonic kidney, and identified *Sall1* binding sites throughout the genome^20^. We re-analysed these data and found that the *Sall1* protein bound to the upstream regions of the *Decorin* and *Fat4* loci (Fig. 6). *Sall1* also bound to many stroma-related gene loci, including *Pbx1*, *Meis1*, *Hoxd10*, and *Tcf21*, but not to *Foxd1* or *Pdgfrβ* (Fig. 6). Despite these *Sall1* bindings, the expression levels of these stroma-related genes were largely unaffected, except for *Decorin* and *Fat4* (Fig. 4, Supplementary Fig. S3). These findings suggest that *Decorin* and *Fat4* are likely to be direct targets of *Sall1* in the kidney.

## Discussion

The epithelial component of the nephron is formed through interactions between nephron progenitors in the metanephric mesenchyme and the ureteric bud. However, the stroma also plays important roles in regulating the development of the other two lineages. We showed previously that *Sall1* is essential for the maintenance of nephron progenitors^20^, but found here that *Sall1* in the stromal progenitors restricted nephron progenitor expansion in a non-cell autonomous manner. Therefore, *Sall1* is essential for both nephron and stromal progenitors, but exerts opposite effects against nephron progenitors. Given that the *Sall1* protein binds to the Mi2/NuRD repressor complex in the developing kidney^20^,^25^, *Sall1* may directly inhibit *Decorin* expression in the stroma*. Decorin* is de-repressed upon *Sall1* deletion in the stroma, which in turn inhibits Bmp-mediated nephron differentiation resulting in progenitor expansion^9^. Because the *Sall1*-binding peak in the proximal promoter of *Decorin* was within 5 kb from the reported Foxd1-binding site^9^, it would be interesting to examine the relationship between *Sall1* and Foxd1 in the regulation of *Decorin*. *Sall1* can also function as an activator^20^, therefore *Fat4* might be activated directly by *Sall1* and restrict nephron progenitor expansion. To prove these hypotheses and examine the relative importance of the two target genes, reducing *Decorin* or increasing *Fat4* expressions *in vivo* in the *Sall1* mutant kidneys should ameliorate the phenotypes.

However, the changes in expression levels of *Decorin* and *Fat4* were mild even when the phenotype became apparent at E14.5. *Sall1* binds to many stroma-related gene loci, which is reminiscent of the situation in nephron progenitors where *Sall1* binds to many key loci, such as *Six2, Osr1, Eya1,* and *Pax2*^20^. Perhaps *Sall1* forms a network with other nuclear factors, both by protein–protein interaction and mutual transcriptional activation/repression, thereby maintaining respective progenitors. In this type of network, deletion of *Sall1* alone would lead to minor changes in many targets, which still lead to characteristic phenotypes. Indeed this holds true for *Sall1* in the maintenance of nephron progenitors and for *Sall4* in embryonic stem cells^20^,^26^,^27^. Alternatively, *Fat4* and *Decorin* might play only subsidiary roles in enhancing the phenotype caused by other yet-to-be-identified *Sall1* targets. Comprehensive microarray analysis of the mutant stromal cells would help in identifying such targets.

The mechanism underlying the dilatation of nephrons in *Sall1* mutants remains unclear. Because proliferation or survival of the E-cadherin-positive nephron epithelia were not significantly affected at birth (Supplementary Fig. S2), other mechanisms, including impaired planar cell polarity, might be involved. Nephron epithelial cells usually divide along the elongating axis, but alteration of the dividing orientation could lead to increased diameter. Because *Foxd1* deletion accompanied by *Decorin* up-regulation does not produce this phenotype, *Decorin* might not be involved primarily in nephron dilatation in the *Sall1* mutants. Ubiquitous *Fat4* deletion produces a similar phenotype caused by impaired planar cell polarity^14^. However, it needs to be determined whether nephron dilatation is caused by the absence of *Fat4* in the nephron or in the stroma, because *Fat4* expression in the *Sall1* mutants was reduced only in the stroma, but not in the nephron. A collecting duct-specific *Wnt9b* deletion also produces a similar phenotype of nephron dilatation^28^, but it remains unclear whether *Wnt9b* exerts its planar polarity effect to the nephron epithelia in an autocrine manner or by way of the stroma. Therefore, it is possible that stromal genes regulated by *Sall1* mediate the planar polarity effect evoked by *Fat4* or *Wnt9*. Identification of such *Sall1* targets will be important to reveal the unappreciated role of the stroma.

In summary, *Sall1* expression in stromal progenitors non-cell autonomously restricted excessive nephron progenitor expansion and regulated nephron diameter. The former function might be mediated by *Decorin* and *Fat4*, at least partially. Because *Sall1* mutations are found in human hereditary diseases^16^, our finding will also be useful for clinical paediatrics. Furthermore, we have recently succeeded in inducing Six2/*Sall1*-positve nephron progenitors and immature nephron tissues from mouse embryonic stem cells and human induced pluripotent cells^24^. However, stromal cells are required to generate the fully organised kidney. Further studies on the stromal cells, as well as their interactions with nephrons, would help advance our understanding toward the generation of the complex three-dimensional structures of the kidney.

## Methods

### Generation of conditional *Sall1* mutant mice

*Sall1*^*flox*^ mice were produced as described^20^,^27^. *Foxd1GFPCre* (012463) and *R26R-tdTomato* (007905) mice were obtained from the Jackson Laboratory^3^,^29^. The primers used for genotyping were as follows: Cre1 (5′–AGGTTCGTTCACTCATGGA–3′) and Cre2 (5′–TCGACCAGTTTAGTTACCC–3′) for the *Cre* allele (250 bp); *Sall1* flox2 (5′–CCTCTGCCCGAGAGATCG–3′), *Sall1*flox3 (5′–GGCGCGTCTGATTTTATTTC–3′) for the *Sall1* allele (wild-type: 220 bp; mutant: 280 bp). Polymerase chain reaction (PCR) amplifications were performed using GoTaq DNA polymerase (Promega) by denaturation at 95 °C for 2.5 min, followed by 35 cycles of 95 °C for 30 s, 58 °C for 60 s, and 72 °C for 30 s, and a final extension at 72 °C for 7 min. All animal experiments were performed in accordance with the institutional guidelines and approved by the licencing committee of Kumamoto University (#A27-018).

### Immunohistochemistry

Tissues were fixed in 10% formalin, embedded in paraffin wax and cut into 6-μm sections. Immunostaining was carried out automatically using a BlueMap kit and the Discovery System (Roche) or manually for immunofluorescence staining. The following primary antibodies were used: anti-*Sall1*^26^ (PPMX Perseus Proteomics: PP-K9814-00); anti-Six2 (Proteintech); anti-Six2 (Abnova); anti-Wt1 (Santa Cruz); LTL (Vector); anti-Slc12a3 (Millipore); anti-cytokeratin (Sigma-Aldrich); anti-Ncam (Developmental Studies Hybridoma Bank #5B8); anti-E-cadherin (BD Biosciences: mouse-derived, Cell Signaling: rabbit derived), anti-CD31 (Abcam); anti-Pdgfrβ (Cell Signaling); anti-Aldh1a2 (Abcam); anti-PHH3 (Millipore; rabbit-derived, Abcam; mouse-derived); anti-Red Fluorescent Protein (RFP; Rockland); and anti-GFP (Abcam; chicken-derived). In paraffin sections, no GFP or tdTomato signals were detected unless the respective antibodies were used. TUNEL assays were performed using an ApopTag Plus fluorescein *in situ* apoptosis detection kit (Millipore), and the signal was enhanced using the biotin-conjugated anti-digoxigenin antibody (Sigma-Aldrich) and Alexa 594-conjugated streptavidin (Life Technologies). Immunofluorescence was visualised with an LSM780 confocal microscope (Zeiss).

### Quantification of immunostained samples

Six2-positive nephron progenitors surrounding the ureteric bud were counted from the sections of four control and six mutant kidneys at E12.5, and from six kidneys for each genotype at E14.5. The Six2 images in green colour channel were incorporated into Image J^30^, trimmed, and subjected to smoothing for noise elimination, before cell counting. Numbers of PHH3- or TUNEL-positive cells out of Aldh1a2-positive stromal cells or E-cadherin-positive nephron epithelia were counted manually using high-resolution images from sections of six kidneys for each genotype at E13.5, 14.5, and P0. Data were evaluated for statistical significance using Student’s t-test.

### *In situ* hybridisation

For whole-mount *in situ* hybridisation, embryonic kidneys were fixed with 4% paraformaldehyde and processed using an automated InsituPro VS (Intavis AG) according to the manufacturer’s protocol. Templates for the probes were generated by reverse transcription (RT)-PCR and sequenced. Multiple pairs of control and mutant kidneys (for example, 4 pairs for *Foxd1*) were examined. *In situ* hybridisation was performed using paraffin sections and an automated Discovery System (Roche) according to the manufacturer’s protocols.

### Quantitative RT–PCR from sorted stromal cells

The new-born kidneys were dissociated into single cells by using a mixture of collagenase XI (Sigma-Aldrich), dispase (Life Technologies), and DNase (Roche) at 37 °C for 10 min and subsequent treatment with 0.25% trypsin-EDTA at 37 °C for 5min. Cells were stained with an Allophycocyanin-conjugated anti-Pdgfrβ antibody (Biolegend) and a biotin-conjugated anti-integrin α8 antibody (R&D Systems), followed by staining with the Phycoerythrin-conjugated streptavidin. Pdgfrβ+/integrin α8− stromal cells, Pdgfrβ−/integrin α8+ nephron progenitors, and the remaining Pdgfrβ−/integrin α8− cells were sorted using a FACSAria SORP (BD Biosciences). RNA was isolated using an RNeasy Plus Micro Kit (Qiagen) and reverse-transcribed with random primers using the Superscript VILO cDNA Synthesis Kit (Life Technologies). Quantitative PCR was carried out using the Dice Real Time System Thermal Cycler (Takara Bio) and Thunderbird SYBR qPCR Mix (Toyobo). Primer sequences are shown in Supplementary Table. All the samples were normalized against β-actin expression.

### ChIP-Seq analysis

ChIP-Seq analysis of the whole kidneys at E16.5 was performed as described previously^20^, and the data have been deposited to Genbank/DNA Data Bank of Japan (accession no. DRA000957). The sequences were mapped to the mouse genome (mm9).

## Additional Information

**How to cite this article**: Ohmori, T. *et al.* *Sall1* in renal stromal progenitors non-cell autonomously restricts the excessive expansion of nephron progenitors. *Sci. Rep.* **5**, 15676; doi: 10.1038/srep15676 (2015).

## Supplementary Material

Supplementary Information
