# Supplementary material for: Sall1 in renal stromal progenitors non-cell autonomously restricts the excessive expansion of nephron progenitors
Source: Sci Rep. 2015 Oct 29;5:15676. doi: 10.1038/srep15676 (PMC4625151; doi:10.1038/srep15676)

Supplemental information

Sall1 in renal stromal progenitors non-cell autonomously restricts the excessive expansion of nephron progenitors

Tomoko Ohmori, Shunsuke Tanigawa, Yusuke Kaku, Sayoko Fujimura, and Ryuichi Nishinakamura*

Department of Kidney Development and Liaison Laboratory Promotion Facility, Institute of Molecular Embryology and Genetics, Kumamoto University, Kumamoto 860-0811, Japan

*** E-mail: ryuichi@kumamoto-u.ac.jp**

Supplementary Figure S1 │ Sall1 expression in the stroma is absent in the *Foxd1GFPCre; Sall1$^{flox/flox}$* mice.

Immunostaining for Sall1, Six2, and GFP of *Sall1$^{flox/flox}$*, *Foxd1GFPCre; Sall1$^{flox/+}$*, and *Foxd1GFPCre; Sall1$^{flox/flox}$* kidneys at E12.5 (A) and E14.5 (B). Serial sections of each genotype were stained. Sall1 in the stroma (asterisks) is absent, while GFP indicating *Foxd1* expression is retained in the homozygous mutant. Sall1 is not expressed in the medullary regions at E14.5. ub: ureteric bud. Scale bars = 100 μm

Supplementary Figure S2 │ Survival, proliferation, and lineage commitment of the stromal progenitors are not affected in the *Sall1* mutants.

(A) TUNEL and Aldh1a2 staining of *Sall1$^{flox/flox}$* and *Foxd1Cre; Sall1$^{flox/flox}$* kidneys at E14.5.

(B) The percentages of TUNEL-positive cells in the Aldh1a2-positive cortical stromal cells at E13.5 and E14.5.

(C) PHH3 and Aldh1a2 staining of *Sall1$^{flox/flox}$* and *Foxd1Cre; Sall1$^{flox/flox}$* kidneys at E14.5.

(D) The percentages of PHH3-positive cells in the Aldh1a2-positive cortical stromal cells at E13.5 and E14.5.

(E) tdTomato expression in *Foxd1Cre; R26R-tdTomato* and *Foxd1Cre; Sall1$^{flox/flox}$*; *R26R-tdTomato* kidneys at E15.5, immunostained using an anti-RFP antibody. Arrows: glomeruli.

(F, G) Survival and proliferation of the neonatal nephron epithelia are not affected in the

Sall1 mutants. The percentages of TUNEL-positive (F) or PHH3-positive (G) cells are shown for the E-cadherin-positive nephron epithelia in the cortex and medulla.

Scale bars: (A, C) = 20 μm; (E) = 100 μm. Results are presented as the mean ± standard error. NS: not significant statistically ($p>0.05$).

Supplementary Figure S3 │ Expression of stromal genes in the control and *Sall1* mutant kidneys.

(A-C) Whole mount *in situ* hybridisation of the embryonic kidney at E14.5. The expression levels of *Meis1* (A), *Hoxd10* (B), and *Tcf21* (C) are unaffected. *Meis1* and *Hoxd10* are also expressed in the neighbouring nephron progenitors, while *Tcf21* is expressed in the medullary stroma located deep inside the tissue. Therefore, the border of the expression domain is somewhat vague. Scale bars = 100 μm.

Supplementary Table │ Primer sequences used for qPCR.

Primers for quantitative RT-PCR		
Gene	Sequence (5' - 3')	
Aldh1a2	F	AGCCCATTGGAGTGTGTGGAC
	R	TGCTCAGCGGGTTTGATGAC
β-Actin	F	CATCCGTAAAGACCTCTATGCCAAC
	R	ATGGAGCCACCGATCCACA
Decorin	F	CTGGGCTGGCACAGCATAAGTA
	R	GAACGCACATAGACACATCTGAAGG
Fat4	F	GTGTTTAACGTCACCGATGCAGA
	R	TCCACTGCAAACTGCCCAAG
Pdgfrβ	F	GAACGACCATGGCGATGAGA
	R	GCATCGGATAAGCCTCGAACA
Six2	F	GCAACTTCCGCGAGCTCTAC
	R	GCCTTGAGCCACAACTGCTG

Supplementary Figure S1

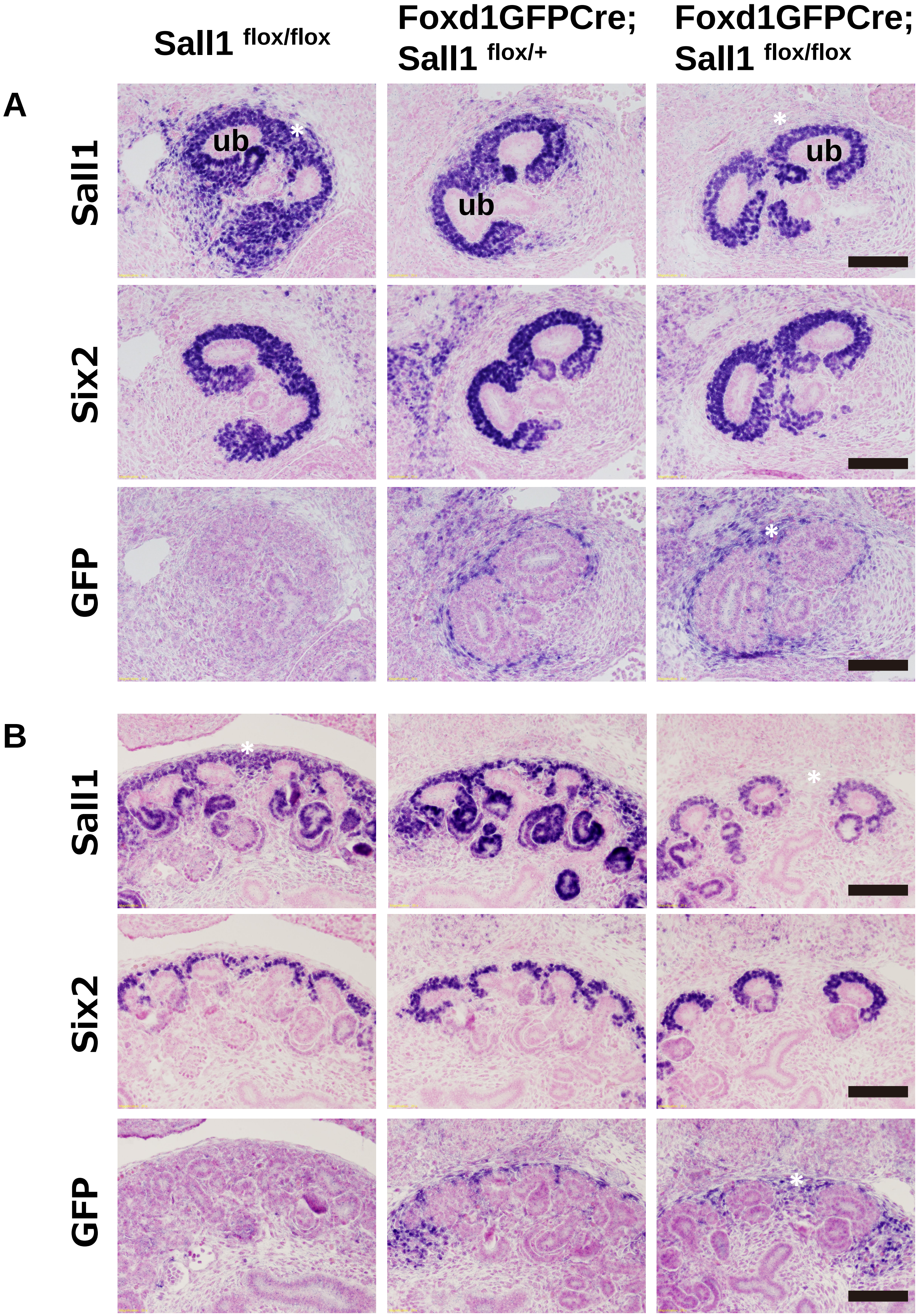

Supplementary Figure S2

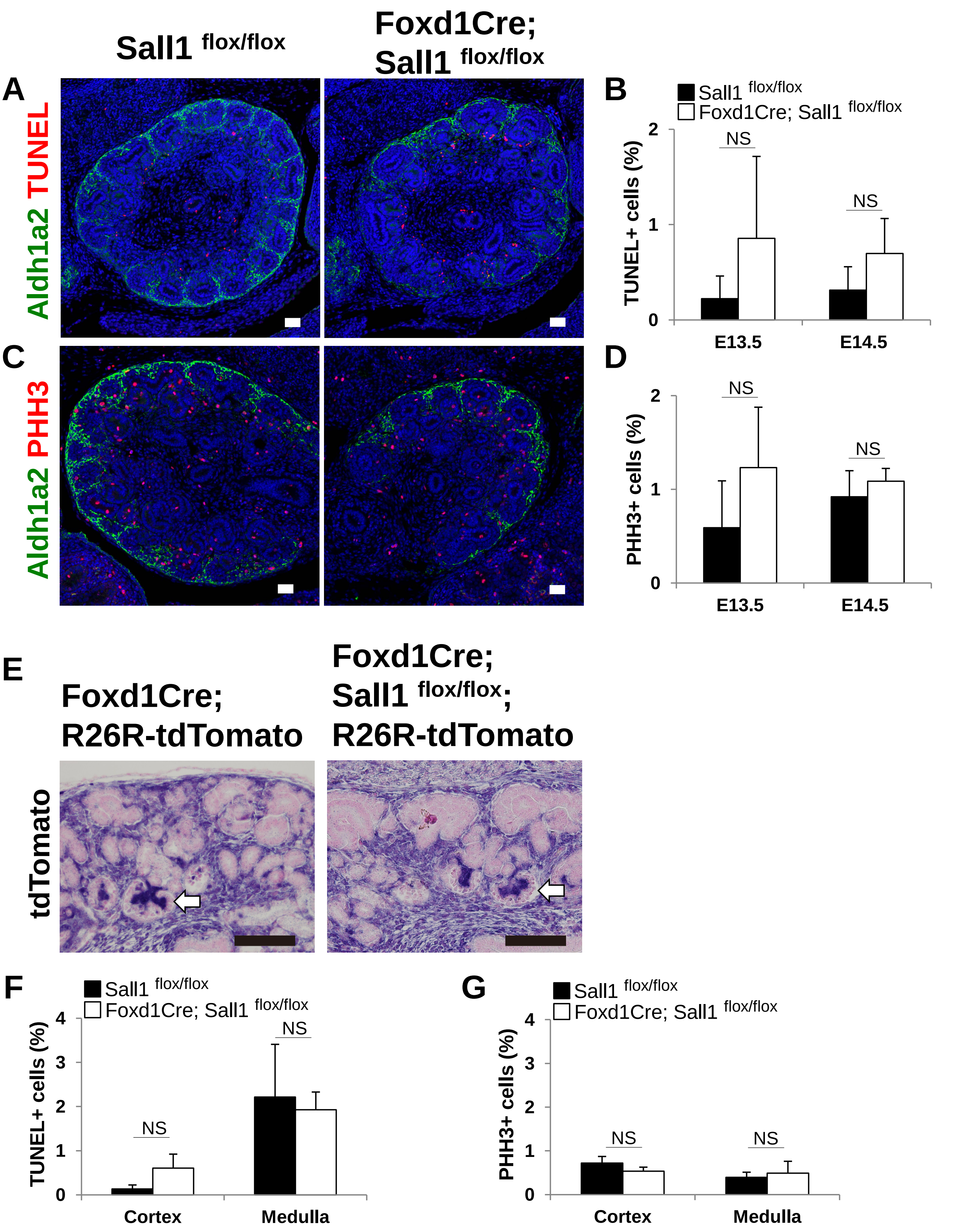

Supplementary Figure S3

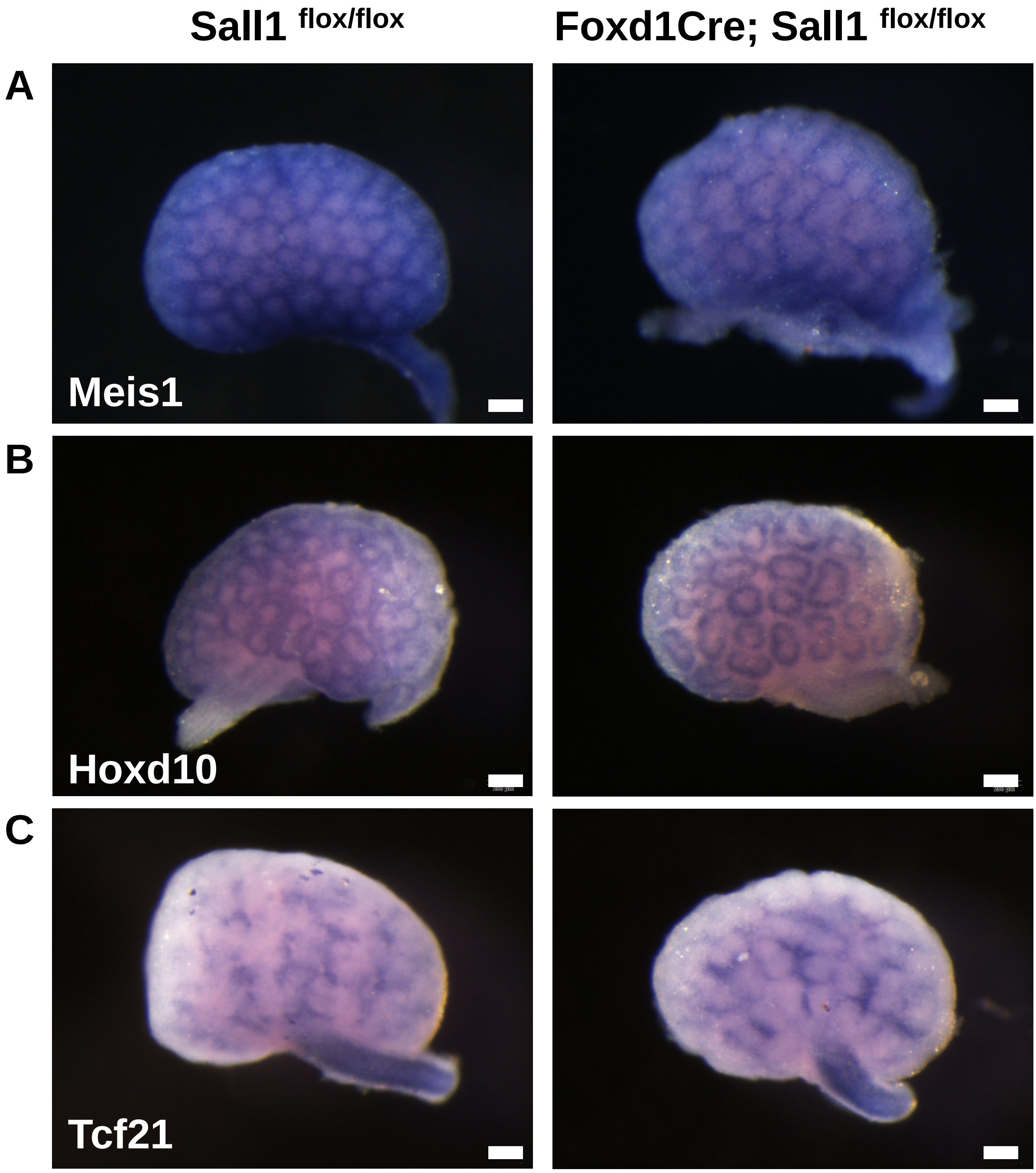

Supplement: Supplementary Information [file srep15676-s1.pdf]
